# Six transition patterns and seven capture types in different left bundle branch bipolar pacing configurations

**DOI:** 10.3389/fcvm.2024.1430529

**Published:** 2024-09-04

**Authors:** Lu Zhang, Longfu Jiang, Binbin Luo, Jiabo Shen, Hao Wu, Weifang Zeng

**Affiliations:** ^1^ Department of Cardiology, Ningbo No. 2 Hospital, Ningbo Cardiovascular Center, Ningbo, Zhejiang, China; ^2^ Cardiovascular Disease Clinical Medical Research Center of Ningbo, Ningbo, Zhejiang, China

**Keywords:** conduction system pacing, left bundle branch pacing, bipolar pacing, electrophysiological mechanism, physiological pacing

## Abstract

**Aims:**

This study aims to explore the different transition patterns and capture types during two bipolar pacing tests based on the selective left bundle branch (LBB) capture determined by the continuous pacing and recording technique.

**Methods:**

In total, 67 patients completed two unipolar and two bipolar pacing tests based on selective LBB capture during screwing-in for left bundle branch pacing (LBBP) using the continuous pacing and recording technique. The electrophysiological characteristics and potential mechanisms of different pacing configurations were further evaluated in this study.

**Results:**

We found six transition patterns and derived seven capture types in two bipolar pacing tests according to the analysis of continuous electrocardiogram and electrogram changes. Compared with the conventional configuration of “Tip−Ring+” bipolar pacing, “Ring−Tip+” testing had a lower threshold for simultaneous capture of the LBB and the left and right ventricular septum myocardium (1.57 vs. 2.84 V at 0.5 ms) and was the only configuration to yield the peculiar “LBBP + right ventricular septum pacing (RVSP)” capture type.

**Conclusions:**

In this study, we observed for the first time that “Ring−Tip+” bipolar pacing allows for a lower clinically applicable pacing threshold for simultaneous capture of the LBB and left and right ventricular septum myocardium, and the peculiar “LBBP + RVSP” capture type. This may be a more advantageous physiological pacing configuration, warranting further investigation and application in the future.

**Lay summary:**

Based on the specific selective LBB capture, we first found six transition patterns and derived seven capture types in two bipolar pacing tests due to the different thresholds of the LBB, left ventricular septal myocardial, and right ventricular septal myocardial. Compared with the conventional configuration of “Tip−Ring+” bipolar pacing, “Ring−Tip+” testing had a lower threshold for simultaneous capture of the LBB and the left and right ventricular septum myocardium (1.57 vs. 2.84 V at 0.5 ms) and was the only configuration to yield the peculiar “LBBP + RVSP” capture type. More pacing strategies should be released and investigated to achieve the best physiological pacing according to the individualized electrophysiological characteristics of patients.

## Introduction

Left bundle branch pacing (LBBP) is a novel procedure in the field of physiologic pacing, however, the traditional intermittent pacing technique does not allow for the continuous monitoring of the electrocardiogram (ECG) and electrogram (EGM) to diagnose the capture of the left bundle branch (LBB) due to the limitation of connecting through an alligator clamp(1, 2). In addition, improper high-pass filter (HPF) settings on a discrete local ventricular electrogram also limit the selective LBBP (SLBBP) acquisition rate, which was only 26.4%–41% in previous studies (3, 4). However, our center achieved a higher SLBBP rate (84.5%–91%) according to a novel implantation technique based on continuous pacing and monitoring of the ECG and EGM and a modified HPF setting (200/500 Hz) (5, 6). In the context of specific capture of the LBB, Wu et al. (7) clearly described non-seletive (NS)-bipolar-left bundle (LB), NS-cathodal-LB, S-cathodal-LB, and left ventricular septal (LVS)-cathodal in conventional “Tip-Ring+” testing, which represented the capture types of left ventricular septal myocardial (LVSM) + right ventricular septal myocardial (RVSM) + LBB,” “LVSM + LBB,” “SLBBP,” and “selective left ventricular septal pacing (SLVSP).” This inspired us to investigate whether there will be more capture types and added the “Ring–” (ring electrode as the cathode) and “Ring–Tip+” (ring electrode as the cathode and tip electrode as the anode) threshold tests from 3 March 2022, and then a new “RVSM + LBB” capture type was discovered (8).

In this study, we explore the different transition patterns and capture types by decreasing the output in two kinds of bipolar pacing in the background of clear selective LBB capture and attempt to evaluate their electrophysiological characteristics and potential mechanisms.

## Methods

### Study participation

From March 2022 to December 2022, a total of 72 (88.9%) patients achieved selective LBB capture among the 81 patients who underwent the LBBP procedure in this study. Three of them had a dislocation of the lead while removing the sheath and two did not perform the “Ring–” and “Ring–Tip+” threshold tests because they could not tolerate the time of the procedure. Finally, 67 patients completed the two unipolar and two bipolar threshold tests. Patient- and procedure-related characteristics were recorded as well. This study adheres to the Helsinki Declaration and was approved by the ethics committee of Ningbo No. 2 Hospital. Written informed consent was obtained from all the participants.

### Implantation procedure

The LBBP procedure and the cable connection description have been previously published (9) and the relevant articles were cited in the 2023 EHRA clinical consensus statement on conduction system pacing implantation (6). Following left axillary vein or left subclavian vein puncture and connection of the Model 3830 pacing lead (Medtronic, Minneapolis, MN, USA), John Jiang's connecting cable, the C315 His sheath (Medtronic, Minneapolis, MN, USA) was inserted into the right ventricle. The 3830 lead was positioned at the LBBP target site under fluoroscopic guidance and then gradually screwed into the interventricular septum. Guided by this cable, continuous 12-lead ECG and intracardiac EGM were recorded under 2 V/0.5 ms output for all patients from the electrophysiological recording system (EP-Workmate, Abbott Laboratories, Chicago, IL, USA) during LBB lead implantation, and all subsequent test data were recorded and could be analyzed retrospectively. The key points were to get the specific S-V isoelectric interval of the EGM as the endpoint of selective LBBP according to a beat-to-beat analysis of the screwing-in process.

Afterward, we performed four threshold tests before pacemaker implantation by decreasing the output from 8 V to near-threshold value (8): (A) tip electrode as the cathode (Tip−); (B) ring electrode as the cathode (Ring−); (C) tip electrode as the cathode and ring electrode as the anode (Tip−Ring+); (D) ring electrode as the cathode and tip electrode as the anode (Ring−Tip+). During offline analysis, two independent doctors confirmed the dynamic changes in ECG and EGM morphology and measured the intervals without any information on pacing modes. The V1 R-wave peak time (RWPT) and V6 RWPT were measured from the pacing signal to the peak of the R-wave in the lead V1 and V6. The sweep speed for accurate measurement was 200–600 mm/s with appropriate signal augmentation. All information was finally reviewed and verified by a senior physician.

### Statistical analyses

Data management and statistical analyses were performed using SAS software version 9.3 (SAS Institute). All variables were presented as mean ± SD or frequencies and percentages, as appropriate. Student's *t-*test was used for continuous variables and the chi-square (or Fisher's exact) test was applied for categorical data. Statistical significance was a two-sided probability of 0.05 or less.

## Results

A total of 67 individuals were included in our study, with an average screw depth of 14.98 mm and a mean age of 73 years (±9 years), including 33 (49.3%) men and 34 (46.3%) women. For pacing indication, the proportions of atrioventricular block, sick sinus syndrome, atrial fibrillation with bradycardia, and heart failure were 68.6%, 25.4%, 3.0%, and 3.0%, respectively. Furthermore, 12 (17.9%) patients had complete right bundle branch block (RBBB) and 7 (10.4%) had left bundle branch block (LBBB). The other pacing-related parameters are presented in Table 1.

**Table 1:** Clinical characteristics and pacing procedure-related parameters (*n* = 54).

Age (years)	73 ± 9
Male, *N* (%)	33 (49.3)
Pacing indication, *N* (%)	
Atrioventricular block	46 (68.6)
Sick sinus syndrome	17 (25.4)
Atrial fibrillation with bradycardia	2 (3.0)
Heart failure	2 (3.0)
QRS morphology, *N* (%)	
Narrow QRS	48 (71.6)
RBBB	12 (17.9)
LBBB	7 (10.4)
LVEF(%)	62.6 ± 11.1
LBB potential observed, *N* (%)	48 (71.6)
LBB potential to QRS duration (≥ 20 ms), *N* (%)	45 (93.75)
LBB potential to QRS duration (< 20 ms), *N* (%)	3 (6.25)
Lead depth (mm)	14.98 ± 2.62
Screwing time (min)	3.87 ± 3.31
Abrupt shortening of V6 RWPT (ms0	13.94 ± 5.38
Sensing (unipolar) (mV)	9.98 ± 4.92
Impedance (unipolar) (Ω)	719.3 ± 148.1
LBB capture threshold (unipolar), V/0.5 ms	0.55 ± 0.28
LVS capture threshold (unipolar), V/0.5 ms	0.58 ± 0.24
RVS capture threshold (unipolar), V/0.5 ms	0.86 ± 0.44

RBBB, right bundle branch block; LBBB, left bundle branch block; LVEF, left ventricular ejection fraction; LBB, left bundle branch; RWPT, R-wave peak time; LVS, left ventricular septal; RVS, right ventricular septal.

Values are given as mean ± SD or *n* (%).

### Categorization of various patterns of capture during bipolar pacing

**Figure 1:**
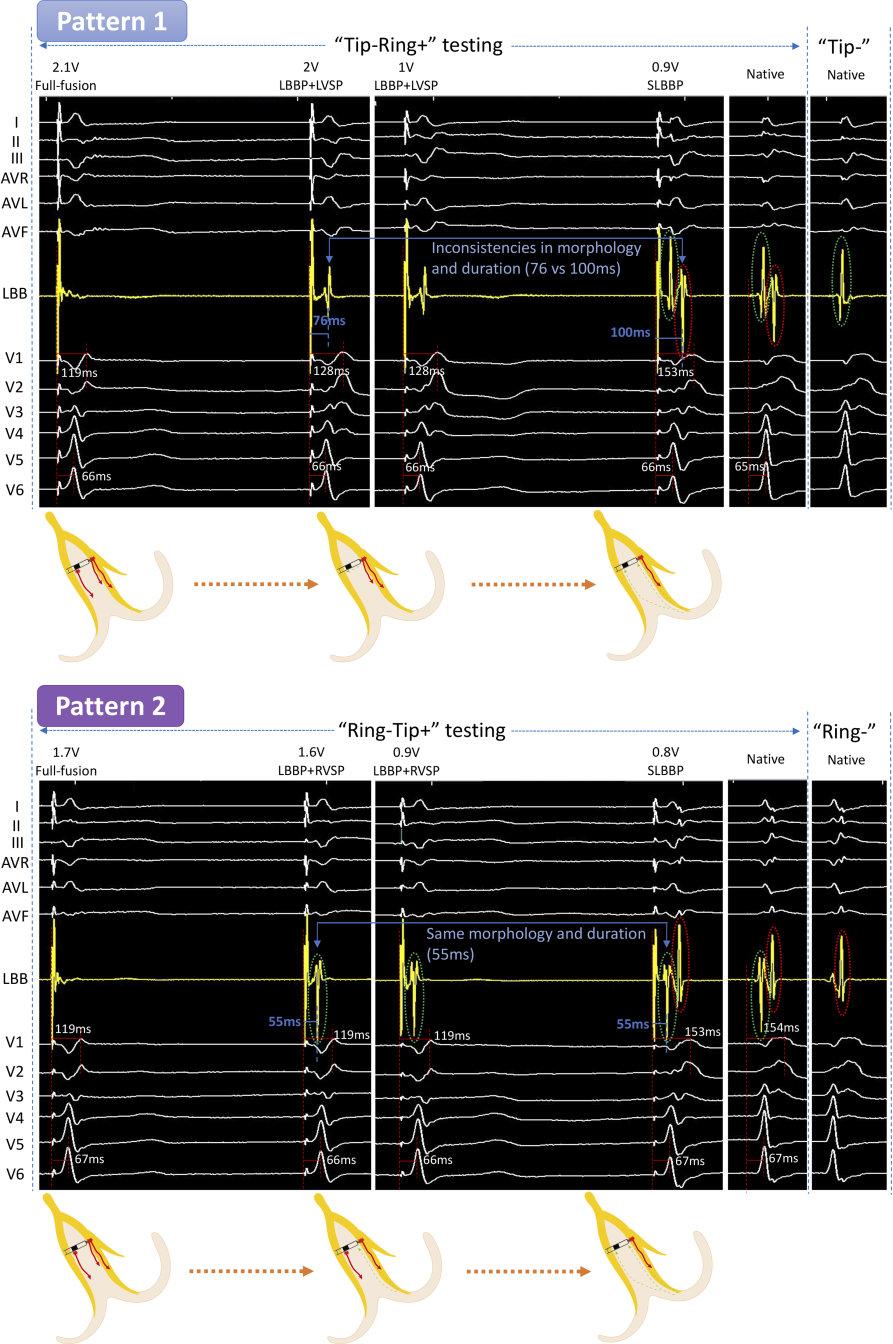
Unedited continuous changes in ECG and EGM for case 1 with complete right bundle branch block. In “Tip−Ring+” testing, the LB-lead showed an independent potential and the V1 RWPT was prolonged at 2 V/0.5 ms output. We speculated that the formation of this independent potential was due to the RVSM lost capture. At 0.9 V/0.5 ms output, the V1 RWPT was further extended and the LB-lead showed two independent waves, which were the same as the intrinsic EGMs. This suggested that LVSM further lost capture and changed to SLBBP. This is the “Full fusion to LBBP + LVSP to SLBBP” pattern (Pattern 1). In “Ring−Tip+” testing at 1.6 V/0.5 ms output, the EGM in the LB displayed a separated potential which was in mirror image agreement with the intrinsic EGM of “Tip−” testing (marked with a green circle), but the V1 RWPT, V6 RWPT, and the ECG morphology were constant. Because it was mirrored and consistent with the intrinsic EGM of “Tip−” testing, we believed its formation to be a result of the LVSM lost capture and that it could only be activated through the LBB. At 0.8 V/0.5 ms output, the EGMs in the LB-lead developed into two independent potentials exactly matching the intrinsic EGMs, while simultaneously, the V1 RWPT was significantly prolonged. This indicated that the RVSM had lost capture and was subsequently converted to SLBBP. Thus, Pattern 2 was a “Full fusion to LBBP + RVSP to SLBBP” pattern. ECG, electrocardiograph; EGM, electrogram; LBBP, left bundle branch pacing; LVSP, left ventricular septum pacing; SLBBP, selective left bundle branch pacing; RVSP, right ventricular septum pacing; LBB, left bundle branch; LVSM, left ventricular septum myocardium; RVSM, right ventricular septum myocardium; RWPT, R-wave peak time.

**Figure 2:**
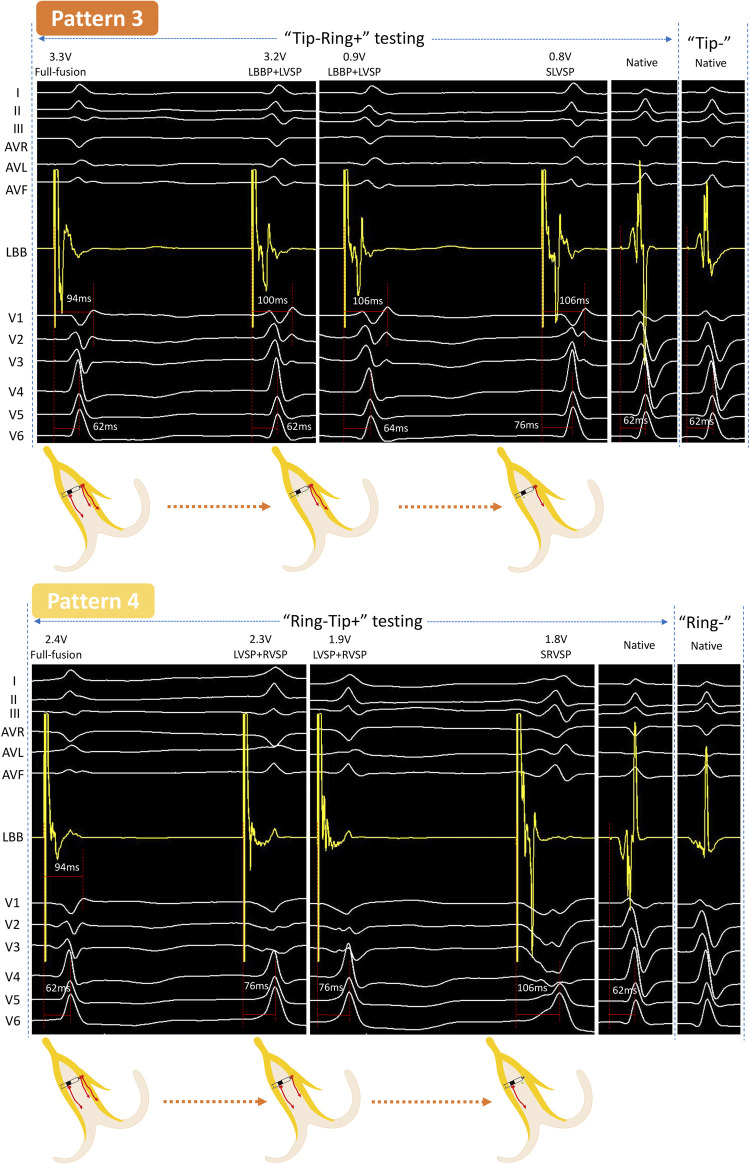
Unedited continuous changes in ECG and EGM for case 2 with normal QRS. In “Tip−Ring+” testing, a new independent waveform appeared in LB-lead at 3.2 V/0.5 ms output with the sudden prolongation of the V1 RWPT, indicating that the RVSM lost capture, while the LVSM and LBB were persistently captured. The EGM was further changed at 0.8 V/0.5 ms and the V6 RWPT was suddenly prolonged, which should be considered as the loss of capture of LBB and conversion to SLVSP. This was a “Full fusion to LBBP + LVSP to SLVSP” pattern (Pattern 3). In “Ring−Tip+” testing, the V6 RWPT abruptly prolonged but without any change to the EGM between two consecutive beats at 2.3 V/0.5 ms, demonstrating that LBB had lost capture and resulted in LVSP and RVSP. At 1.8 V/0.5 ms output, the V6 RWPT further prolonged, so LVSM had lost capture and resulted in SRVSP; the LB-lead presented a wave independent of the pacing signal at the same time. This was a “Full fusion to LVSP + RVSP to SRVSP” pattern (Pattern 4). SLVSP, selective left ventricular septum pacing; SRVSP, selective right ventricular septum pacing. Other abbreviations as in Figure 1.

**Figure 3:**
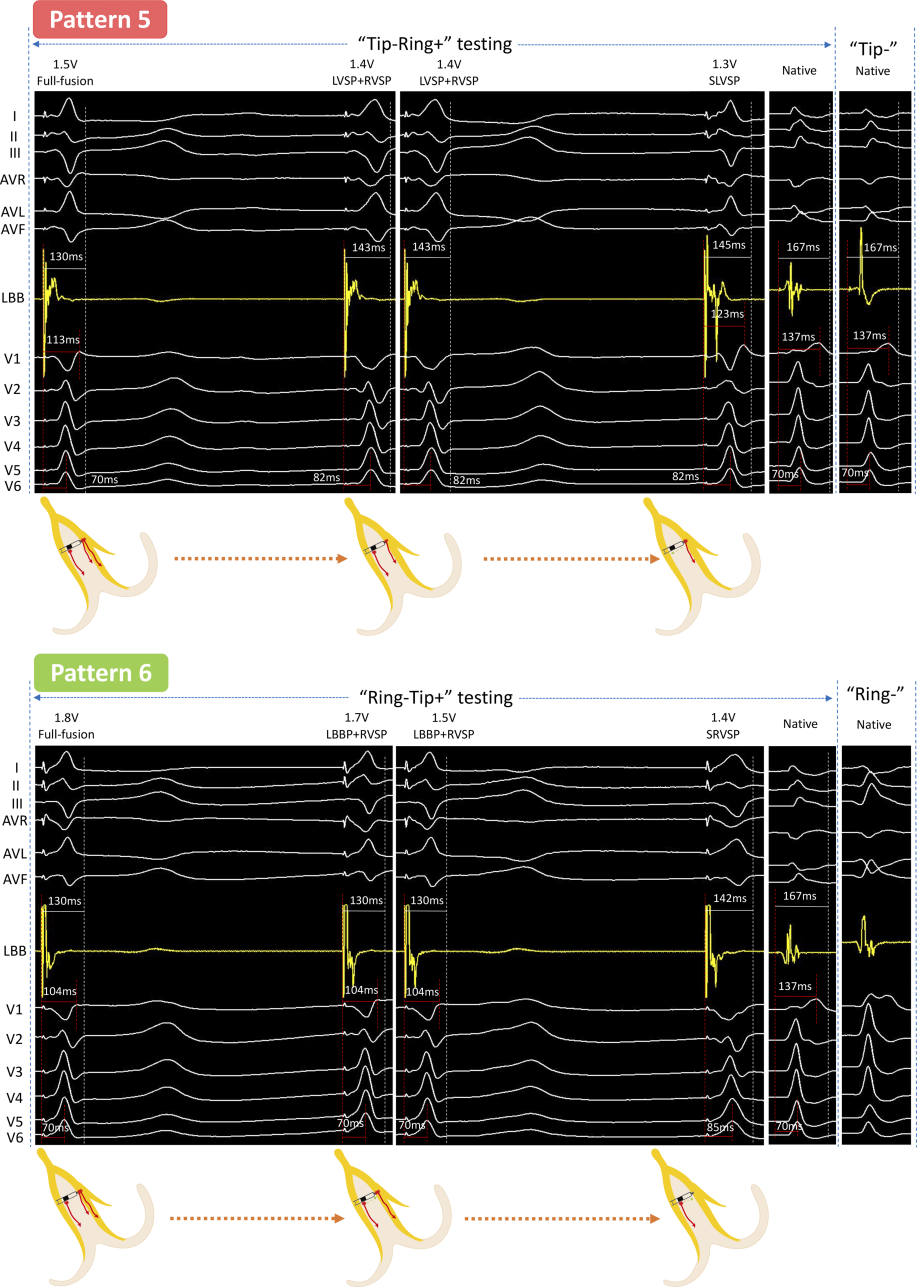
Unedited continuous changes in ECG and EGM for case 3 with complete right bundle branch block. In “Tip−Ring+” testing, the LBB first lost capture at 1.4 V/0.5 ms, which was similar with the changes from “Full fusion to LVSP + RVSP” in Pattern 2. Subsequently, the output was decreased by 0.1 V/0.5 ms, and the EGM presented a new downward amplitude potential accompanied by the RBBB pattern in the ECG, indicating that the RVSM lost capture and need to be activated by the conduction of the LVSM and was thus sensed by the Ring electrode. This was a “Full fusion to LVSP + RVSP to SLVSP” pattern (Pattern 5). In “Ring−Tip+” testing, at 1.7 V/0.5 ms output, a new downward amplitude potential presented at the LB-lead but the V1 and V6 RWPT remained constant; this indicated that the LVSM lost capture. Then the V6 RWPT suddenly prolonged at 1.4 V/0.5 ms output, meaning the LBB further lost capture and resulted in SRVSP. This was a “Full fusion to LBBP + RVSP to SRVSP” pattern (Pattern 6). Abbreviations as in Figures 1, 2.

Among the 67 patients, we found six transition patterns and seven capture types during bipolar pacing threshold testing, which were illustrated by the ECG and EGM changes between two successive heartbeats of the three cases discussed below. All bipolar pacing tests were performed on the basis of specific selective capture of the LBB during the screwing-in process. The bipolar pacing tests for these three cases were presented as Figures 1–3, respectively.

### Case 1 (complete RBBB)

#### **“**Tip−Ring+” testing

The LB-lead showed the fusion wave at high output but showed independent potential separated from the pacing artifact, and the ECG of V1 to V6 presented significant changes when the output was dropped to 2 V/0.5 ms. The V1 RWPT was prolonged from 119 to 128 ms. After further reducing the output to 0.9 V/0.5 ms, the V1 RWPT was further extended to 153 ms and the LB-lead showed two independent waves, which were the same as the intrinsic EGMs. This was the first transition pattern (Pattern 1).

#### “Ring−Tip+” testing

When the output was dropped from 1.7 to 1.6 V/0.5 ms, the EGM in LB displayed a separated potential which was in mirror image agreement with the intrinsic EGM of “Tip−” testing (marked with a green ring), but the V1 RWPT, V6 RWPT, and ECG morphology were constant. At 0.8 V/0.5 ms output, the EGMs in the LB-lead became two independent potentials separated from the pacing signal, which was the same as the intrinsic EGMs. Simultaneously, the V1 RWPT was significantly prolonged from 119 to 153 ms. This was the second pattern (Pattern 2).

### Case 2 (normal rhythm)

#### “Tip−Ring+” testing

When decreasing the output from 3.3 to 3.2 V/0.5 ms, a discrete potential appeared in the LB-lead with a sudden extension (94–100 ms) of the V1 RWPT and a constant V6 RWPT, while the ECG of V1 to V3 showed obvious changes. Furthermore, the V6 RWPT was suddenly prolonged from 64 to 76 ms at 0.8 V/0.5 ms, V1 RWPT was constant, and the EGM was completely different from the intrinsic. This was the third pattern (Pattern 3).

#### “Ring−Tip+” testing

At first, the V6 RWPT was abruptly prolonged for 14 ms in two consecutive beats at 2.3 V/0.5 ms, but there was no significant change in the EGM. Afterward, the V6 RWPT was prolonged for another 30 ms at 1.8 V/0.5 ms, while the LB-lead presented a downward towering wave independent of the pacing signal. This was the fourth pattern (Pattern 4).

### Case 3 (complete RBBB)

#### “Tip−Ring+” testing

At 1.4 V/0.5 ms output, the V6 RWPT was abruptly prolonged from 70 to 82 ms. Next, when the output was decreased by 0.1 V/0.5 ms, V1 showed RBBB morphology and the EGM presented a new downward amplitude potential independent of the pacing signal. This was the fifth pattern (Pattern 5).

#### “Ring−Tip+” testing

At 1.7 V/0.5 ms output, the EGM presented a new downward amplitude potential independent of the pacing signal but the ECG morphology and the RWPT in the V1 and V6 leads remained constant. Next, the V6 RWPT was suddenly prolonged by 15 ms at 1.4 V/0.5 ms output. This was the sixth pattern (Pattern 6).

**Figure 4:**
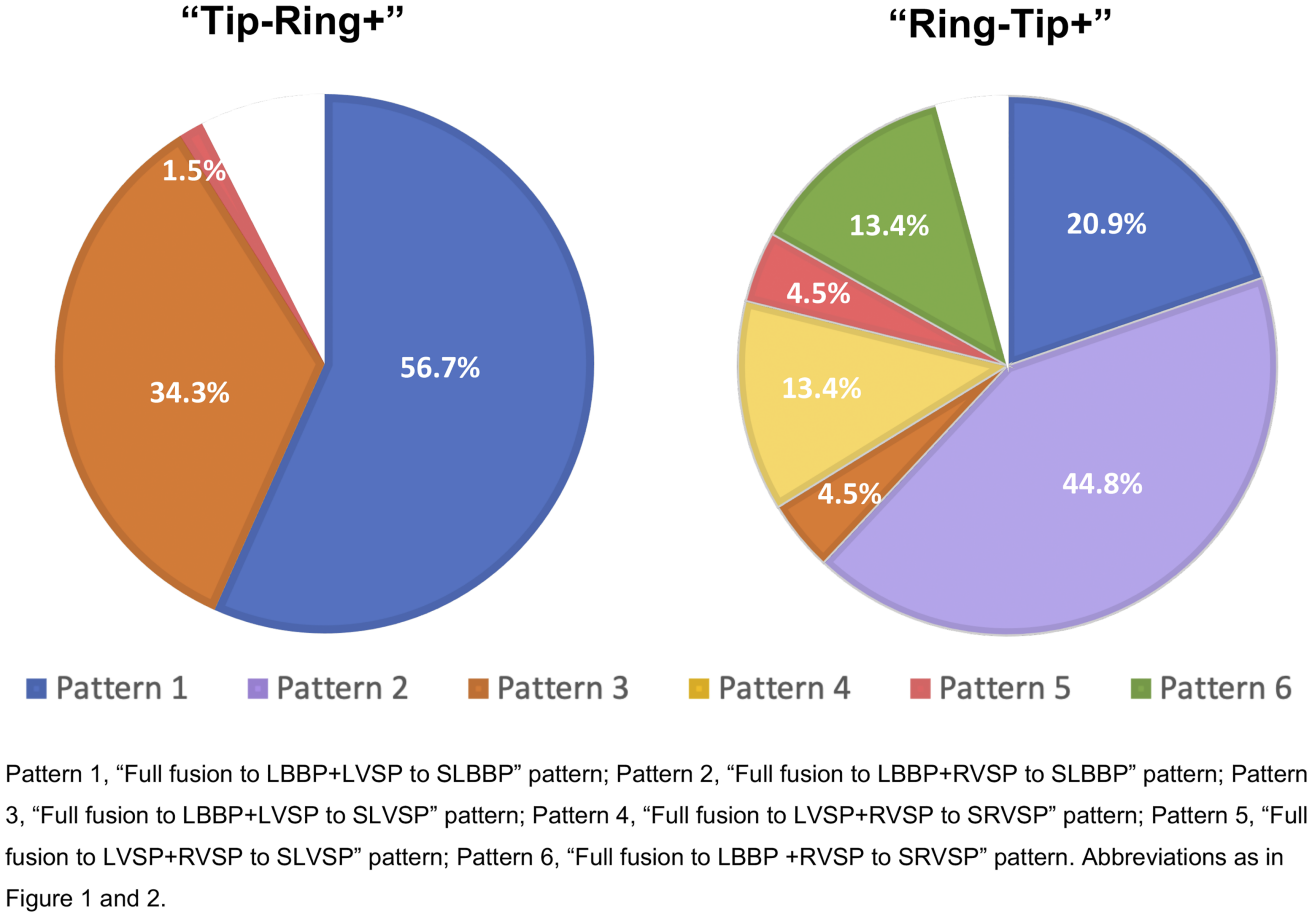
The distribution of six transition patterns in two bipolar pacings. There were only transition Patterns 1, 3, and 5 in “Tip−Ring+” testing, while “Ring−Tip+” testing presented all six transition patterns. The blank areas indicated that there were seven (10.4%) patients in “Tip−Ring+” testing and three (4.5%) participants in “Ring−Tip+” testing for whom the transition modes could not be distinguished due to two of the three components, LBB, LVSM, and RVSM, having identical or similar thresholds. Abbreviations as in Figures 1, 2.

We further summarized the distribution of the six transition patterns in two bipolar pacings (Figure 4). Only transition Pattern 1 (56.7%), Pattern 3 (34.3%), and Pattern 5 (1.5%) were found in “Tip−Ring+” testing, while “Ring−Tip+” testing presented all six transition patterns, accounting for 20.9%, 44.8%, 4.5%, 13.4%, 4.5%, and 13.4%, respectively. In “Tip−Ring+” testing and “Ring−Tip+” testing, we were unable to distinguish transition patterns in seven (10.4%) and three (4.5%) participants, respectively, because two of the three components, LBB, LVSM, and RVSM, had similar or the same thresholds.

## Discussion

The diagnostic “gold standard” LBB capture is a specific S-V isoelectric interval of EGM, which should be confirmed by dynamic pacing programs (4, 10). However, a preferable success rate of SLBBP is a challenging task because of the limitation of the traditional intermittent pacing technique. Our center achieved an 88.9% SLBBP rate through continuous pacing and the appropriate HPF setting. The average screw depth of the lead was 14.98 mm. We believe that the tip and ring electrodes were placed in the LBB/LVSM and RVSM areas. Due to the different threshold values of the LBB, LVSM, and RVSM, different capture types were bound to appear in the two bipolar configurations. In this study, based on achieving SLBBP during screwing-in, we first summarized all possible transition patterns and capture types in left bundle branch bipolar pacing to analyze the changes of ECG and EGM and attempted to clarify the possible mechanism.

At 8 V/0.5 ms suprathreshold pacing, the LVSM, RVSM, and LBB were activated at the same time, and the LB-lead presented a fusion wave indistinguishable from the pacing signal. By decreasing the output, these three components gradually lost capture and became a two-component capture type and then a selective pacing mode, thus forming six transition patterns and seven capture types. We summarized them as “Full fusion [LBBP + left ventricular septum pacing (LVSP) + right ventricular septum pacing (RVSP)]”, “Semi-fusion (LBBP + LVSP/LBBP + RVSP/LVSP + RVSP)”, and “Selective [SLBBP/SLVSP/Selective RVSP (SRVSP)].” In the context of the specific capture of the LBB and the appropriate HPF setting, the identification of the six patterns was determined by the changes in the ECG and EGM morphology and in the V1 and V6 RWPT. For the local potential generated by the activation of the local myocardium or conduction bundle and sensed by the tip or ring electrode, it would be a fusion wave with a pacing artifact if the activation originated from the pacing signal, while it would be the potential independent of the pacing artifact if the activation originated from the distant myocardium or conduction bundle.

Specifically, we (7) revealed that the full fusion capture type had the shortest V1 RWPT and the narrowest paced QRS (P-QRS) due to the co-activation of RVSM and, sensed by the ring electrode, the EGM showed a fusion wave without any separate potential. In the “LBBP + LVSP” capture type, the loss of RVSP led to the delay of V1 RWPT and the more obvious RBBB morphological changes, and the EGM presented a separate potential which was sensed by the ring electrode but not derived from the pacing signal. For the “LBBP + RVSP” capture type (Pattern 4 in Figure 2 and Pattern 5 in Figure 3), the ECG morphology was highly consistent with that of full fusion, because LBBP was the dominant activation for the left ventricle and LVSP had little influence on the comprehensive QRS. This type must be distinguished by the independent potential in the EGM, which indicated LVSM activation. For the “LVSP + RVSP” capture type, the most significant feature different from full fusion was the extension of V6 RWPT, and the EGM was difficult to distinguish without ECG changes due to the activation sensed by the tip and ring electrodes being consistent with full fusion. The three selective capture types were caused by further output reduction: for SLBBP, the EGM showed double separate potential and the ECG presented RBBB morphology; for SLVSP, the EGM showed separate potential perceived by the ring electrode, and V6 RWPT was prolonged in the ECG; for SRVSP, the ECG mostly presented as the shape of screwing, V6 RWPT was further extended, and the EGM showed an independent potential perceived by the tip electrode.

**Figure 5:**
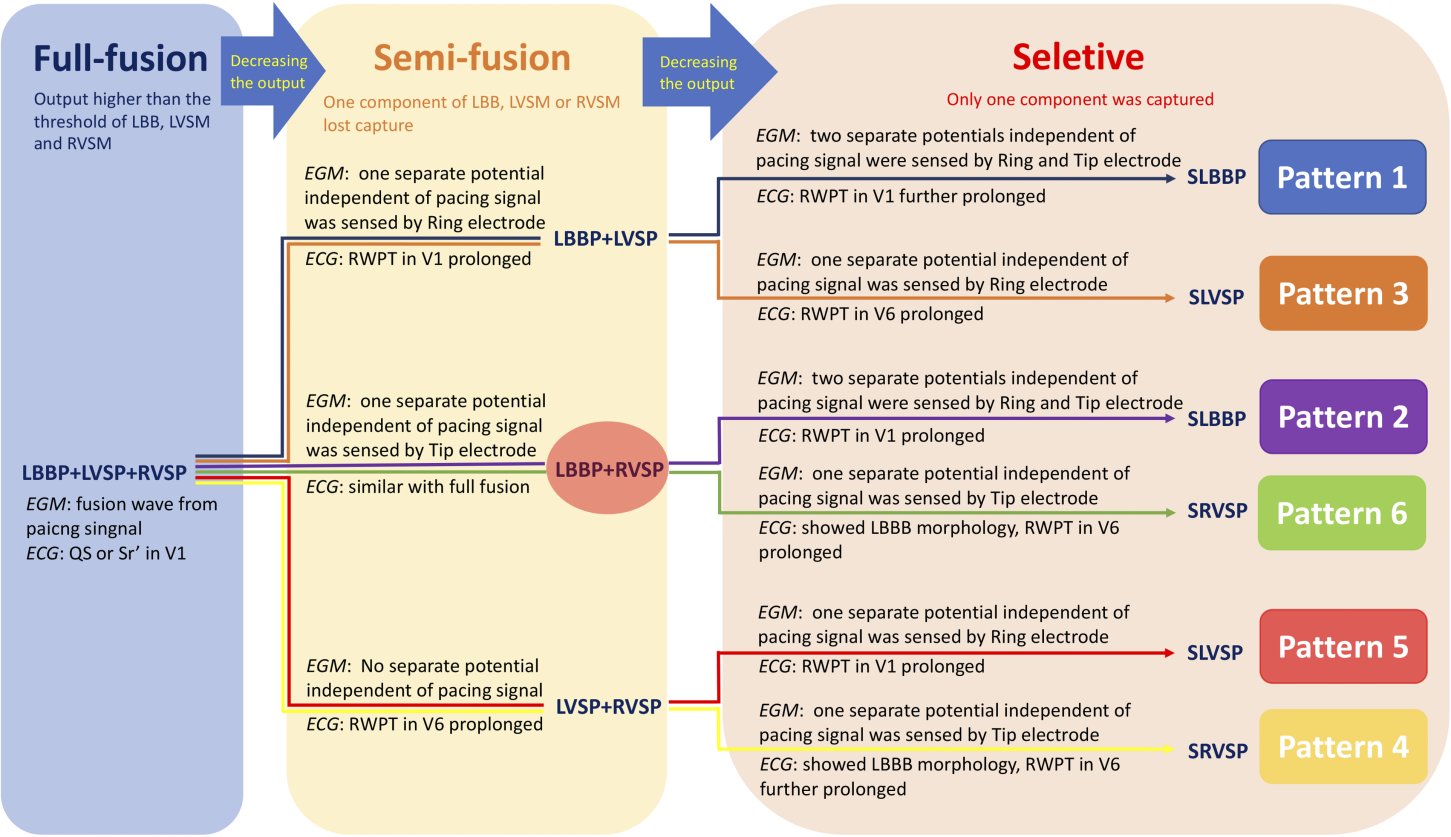
The path diagram of six transition patterns in two bipolar pacings. The six different colored paths corresponded to the six transition patterns in the Figures 1–3. “Full fusion,” “Semi-fusion,” and “Selective” represented the capture of all three components (LBB, LVSM, and RVSM), two components, and only one component, respectively. Abbreviations as in Figures 1, 2.

This is the first time we have proposed six transition patterns and summarized them as a path diagram (Figure 5). Previous scholars have shown that a unipolar EGM can accurately record special morphological characteristics of local myocardial electrical activity, whereas a bipolar EGM is a synthesis of unipolar EGMs from two recording electrodes (11). This theory was verified by comparing intrinsic EGMs of bipolar testing with unipolar testing in Case 1, the first (marked with a green circle) and second waves (marked with a red circle) of intrinsic EGMs in bipolar testing corresponded to the intrinsic EGM of “Tip−” and “Ring−” testing, representing the potential of LVSM and RVSM at the tip and ring electrodes, respectively. Regardless of “Tip−Ring+” or “Ring−Tip+” testing, the EGMs in the SLBBP mode were also consistent with intrinsic EGMs in bipolar testing because they showed that the LVSM and RVSM were both activated by the LBB for this RBBB patient. Moreover, the EGM morphologies were of entire or mirrored consistence due to the different connection between the anode and cathode. For the identification of the source of EGM separation potential after the myocardium at the tip or ring electrode losing capture, it is necessary to compare SLBBP with native EGM morphology, duration, and V1 RWPT, as shown in Pattern 1. In the “LBBP + LVSP” capture type at 2 V/0.5 ms to 1 V/0.5 ms output, the separation potential represented the loss of RVSM and was sensed by the ring electrode. The morphology was different the SLBBP and native EGM of the “Ring−” mode and the S-V interval was shorter (76 vs. 100 ms) than SLBBP (marked as blue arrow) (Pattern 1 in Figure 1). Moreover, V1 RWPT was also shorter than SLBBP (128 vs. 153 ms), so we reasonably speculated that the activation of RVSM was caused by the excitation spreading of LVSP. For the “LBBP + RVSP” to SLBBP capture type (Pattern 2 in Figure 1), the isolated potential in the EGM should be the activation of LVSM spreading by the LBB because its morphology and duration (55 ms) was consistent with SLBBP.

Previous studies have outlined different transition patterns. In a case report, Ponnusamy (12) found the “Double transition sign” during bipolar pacing which would differentiate LBB capture from LVS capture, which is consistent with the first pattern described in our study. Wu et al. (7) clearly described NS-bipolar-LB, NS-cathodal-LB, S-cathodal-LB, and LVS-cathodal, which are equivalent to four capture types from our Patterns 1 and 3. However, these two studies only discussed the changes of the ECG and EGM in “Tip−Ring+” without “Ring−Tip+” testing. In our study, we could easily and accurately distinguish all six transition patterns in “Ring−Tip+” testing, while only Patterns 1, 3, and 5 were observable in “Tip−Ring+” testing. Therefore, it is difficult to find more than two transition patterns in traditional bipolar research.

We calculated the pacing parameters of two bipolar modes according to the different transition patterns (shown in Table 2). All the thresholds of the three components, LBB, LVSM, and RVSM, in the bipolar modes were higher than in the unipolar pacing mode (Table 1). Compared to “Ring−Tip+” testing, “Tip−Ring+” testing had lower LBB (0.6 vs. 0.97 V at 0.5 ms) and LVSM (0.63 vs. 1.33 V at 0.5 ms) thresholds but a higher RVSM threshold (2.84 vs. 1.17 V at 0.5 ms); however, there was no significant difference in sensing and impedance. Moreover, the connection of different cathodes and anodes of the Tip and Ring electrodes seemed to have extra effects on the thresholds of LVSM and RVSM, that is, the local myocardium at the cathode electrode was less affected (LVS: unipolar 0.58 V vs. “Tip−Ring+” 0.63 V vs. “Ring−Tip+” 1.33 V at 0.5 ms; RVS: unipolar 0.86 V vs. “Tip−Ring+” 2.84 V vs. “Ring−Tip+” 1.17 V at 0.5 ms). These might be related to the fact that anodal capture thresholds are known to be higher than cathodal capture thresholds for local myocardium (13), but the specific mechanism remains unclear and further research is required to clarify.

**Table 2:** Parameters of two bipolar pacing threshold testing (*n* = 54).

Characteristics	“Tip−Ring+”	“Ring−Tip+”	*P*-value
Sensing (mV)	14.68 ± 6.54	14.84 ± 6.75	0.89
Impedance (Ω)	740.9 ± 139.1	731.9 ± 116.6	0.69
Threshold (V/0.5 ms)			
Full fusion threshold	2.84 ± 1.15	1.57 ± 0.55	<0.01
LBB + RVS threshold	—	1.01 ± 0.27	—
LBB capture threshold	0.60 ± 0.48	0.97 ± 0.46	<0.01
LVS capture threshold	0.63 ± 0.22	1.33 ± 0.49	<0.01
RVS capture threshold	2.84 ± 1.15	1.17 ± 0.51	<0.01

LBB, left bundle branch; RVS, right ventricular septal; LVS, left ventricular septal.

Values are given as mean ± SD. Full fusion is defined as simultaneous capture of LBB, LVS, and RVS myocardium.

Theoretically, the closest to physiologic pacing among the seven types is the full fusion or LBBP + RVSP capture type, which ensures the capture of the LBB without an interventricular activation delay. A previous study (14) proved that the “Full fusion” type had the shortest V1 RWPT, V6 RWPT, and P-QRS. Lin et al. (15) reported a similar concept, namely, bilateral bundle branch area pacing, which solved the phenomenon of delayed right ventricular activation and was explained by eliminating and diminishing the paced RBBB in the V1 lead, but this study had no exact basis for LBB capture. In addition, it is still uncertain whether the right bundle branch (RBB) could be captured directly because the RBB is too narrow to map and vulnerable to damage by the pacing lead during screwing-in (4). Interestingly, in our study, the “Ring−Tip+” pacing mode required significantly lower output for simultaneous pacing of the LBB, LVSM, and RVSM when compared with the “Tip-Ring+” pacing mode (1.57 vs. 2.84 V at 0.5 ms). To the best of our knowledge, no previous research described the “LBBP + RVSP” capture type. Our study found that this mode only appeared in the “Ring−Tip+” testing, accounting for 52.2% (35/67). The pacing threshold for the “Full fusion” (1.57 V at 0.5 ms) and “LBBP + RVSP” (1.01 V at 0.5 ms) pacing in “Ring−Tip+” testing was still well below the device default setting (typically 2–3 V), indicating that “Ring−Tip+” pacing would not prominently impact pacemaker battery longevity. Of note, the “Ring−Tip+” bipolar pacing mode that was tested during implantation could not be applied in clinical practice because commercially available dual-chamber pacemakers do not allow programming of this pacing configuration. However, this mode should be considered in future for physiological pacing.

Our study had some limitations. First, this study is based on data from a single center; multi-center randomized controlled clinical trials are warranted to verify and explore the underlying mechanism. Second, there should be six transition patterns for “Tip−Ring+” bipolar pacing in theory as well, which may be observed by expanding the sample size. Third, most of the patients in our study had preserved ejection fraction and only a small portion had bundle branch block; therefore, our conclusion is not suitable for patients with cardiomyopathy, who should be included in future in-depth analyses. Fourth, the “Ring−Tip+” bipolar pacing could not be applied in clinical practice at present. Fifth, long-term follow-up is necessary to evaluate the clinical outcomes and adverse events.

The key finding of this study was that there are six transition patterns and seven capture types in bipolar pacing as a result of the different thresholds of LBB, LVSM, and RVSM; the pacing configuration with a lower and clinically applicable threshold (1.01 V at 0.5 ms) for synchronously activating the LBB and RVSM was only in the “Ring−Tip+” mode, which deserves further research in the future. More pacing strategies should be released and investigated to achieve the best physiological pacing according to the individualized electrophysiological characteristics of patients.

## Data Availability

The raw data supporting the conclusions of this article will be made available by the authors, without undue reservation.
