# Supplementary figures and images for: Six transition patterns and seven capture types in different left bundle branch bipolar pacing configurations
Source: Front Cardiovasc Med. 2024 Sep 4;11:1430529. doi: 10.3389/fcvm.2024.1430529 (PMC11409822; doi:10.3389/fcvm.2024.1430529)

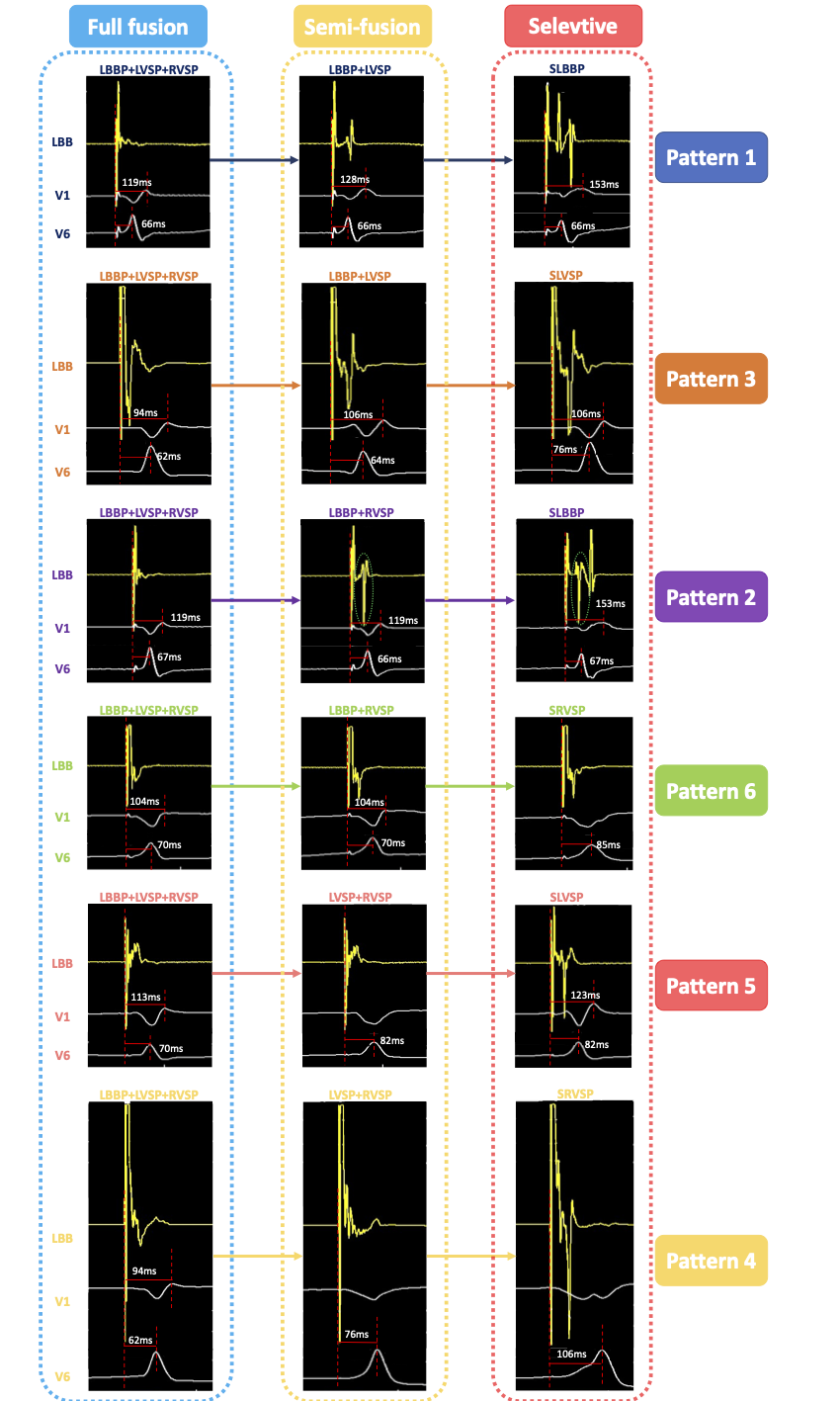

Supplement: Supplementary file 1 [file Image1.tiff]
